# Complete chloroplast genome of medicinal plant *Aconitum carmichaelii*: genome characterization and phylogenetic analysis

**DOI:** 10.1080/23802359.2016.1258342

**Published:** 2016-12-23

**Authors:** Jihai Gao, Dayan Zhang, Wei Wang, Cheng Peng

**Affiliations:** Pharmacy College, Chengdu University of Traditional Chinese Medicine, The Ministry of Education Key Laboratory of Standardization of Chinese Herbal Medicine, Key Laboratory of Systematic Research, Development and Utilization of Chinese Medicine Resources in Sichuan Province-Key Laboratory Breeding Base of Co-founded by Sichuan Province and MOST, Chengdu, China

**Keywords:** *Aconitum carmichaelii*, chloroplast genome, phylogenetic analysis

## Abstract

The complete chloroplast genome sequence of an important medicinal plant of the family Ranunculaceae, *Aconitum carmichaelii* Debx., was characterized in this study. The assembled chloroplast genome was 154,776 bp in length, which included a large single-copy (LSC), a small single-copy (SSC), and two inverted repeat (IR) regions of 86,330bp, 15,986 bp, and 26,193 bp, respectively. The GC content of the genome was 38.1%. Phylogenetic analysis with the whole nucleotide sequences of reported *Aconitum* chloroplast genomes indicated a close relationship of *A. carmichaelii* with *A. kusnezoffii*.

*Aconitum carmichaelii* Debx. belongs to genus *Aconitum* of family Ranunculaceae. *Aconitum* species, which contain various terpenoids, alkaloids, phenypropanoids, and polysaccharides, are well-known in East Asia for their toxicity and as potent herbal medicines (Kadota 1987). Its lateral roots, named as Fuzi, have been extensively used as cardiotonic, analgesic, anti-inflammatory, and diuretic agents to treat colds, polyarthralgia, diarrhoea, heart failure, beriberi, and oedema (Murayama et al. 1991). While the main roots, named as Chuanwu, are used for the clinical treatment of pains and rheumatics (Liu et al. 2012). Due to the similar morphology and drug confusion among *Aconitum* plants, it is very important to carry out identification and phylogeny studies of *A. carmichaelii*. Nineteen *Aconitum* species have been identified using the chloroplast genome intergenic region psb-trnH (He et al. 2010). The phylogenetic relationship of 31 genera of Ranunculaceae have been analyzed applying chloroplast DNA restriction site variation (Johansson 1995). Whereas, more studies on the chloroplast genome are needed for the complete molecular identification. In this study, we characterized the complete chloroplast genome sequence of *A. carmichaelii* to contribute to further molecular and phylogenetical studies of this plant species.

*Aconitum carmichaelii* was collected from Jiangyou GAP (Good Agricultural Practice of Medicinal Plants and Animals) plantation bases of Sichuan province and deposited in The State Bank of Chinese Drug Germplam Resources. The complete chloroplast genome of *A. carmichaelii* was 154,776 bp in size (GenBank accession number KY006977) with 38.1% overall GC content. The genome structure was highly similar to the reported chloroplast genome of closely related species *A. chiisanense* (Lim et al. 2015), and contained 83 protein-coding genes, 35 tRNA genes, and 8 rRNA genes. The chloroplast genome was *composed* of a large single-copy (LSC), a small single-copy (SSC), and two inverted repeat (IR) regions of 86,330bp, 15,986 bp and 26,193 bp, respectively. Thirty-three genes (14 tRNA, 8 rRNA, and 11 protein-coding genes) were duplicated in two inverted repeat regions. As in the chloroplast genomes of other Ranunculaceae species reported (Lim et al. 2015; Park et al. 2015), the *rpl32* gene was lost in *A. carmichaelii*.

A phylogenetic tree of *A. carmichaelii* was constructed based on complete chloroplast genomes of 12 *Aconitum* species by a neighbour-joining analysis using the BLAST pairwise alignments and Blast Tree View tool in NCBI (https://blast.ncbi.nlm.nih.gov/Blast.cgi?PROGRAM=blastn&PAGE_TYPE=BlastSearch&LINK_LOC=blasthome) (Figure 1). The phylogenetic tree indicated that *A. carmichaelii* has a closer genetic relationship with *A. kusnezoffii*, which belongs to the series Inflata Steinb., section Aconitum, subgenus *Aconitum*, as well as our target plant.

**Figure 1.**
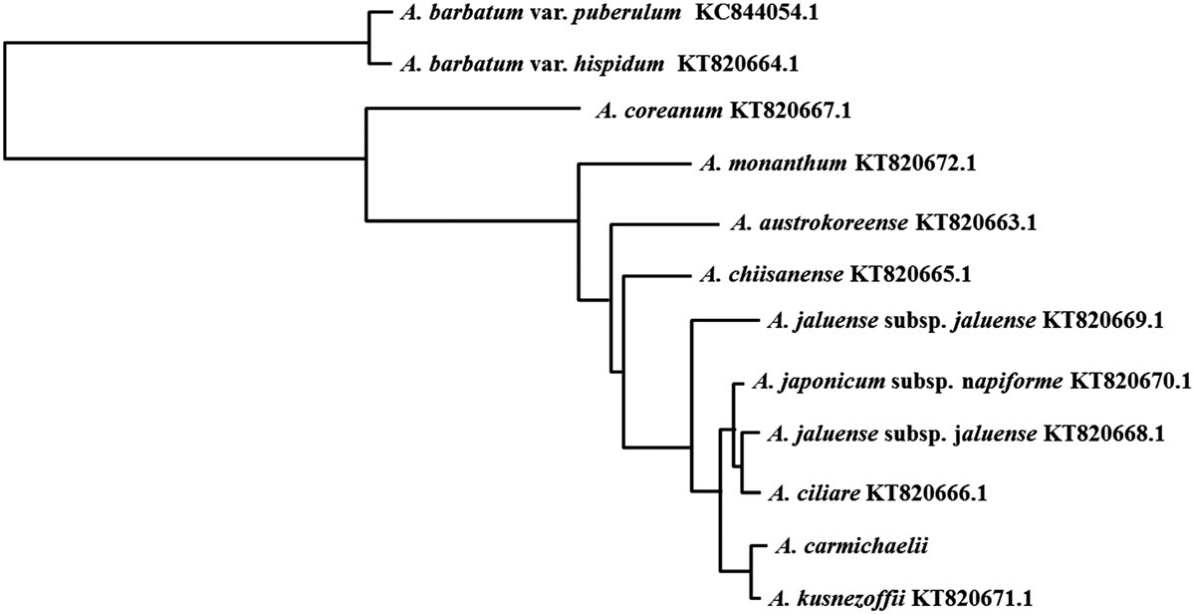
Phylogenetic tree of 12 Aconitum species constructed with the whole chloroplast genomes by a neighbour-joining analysis using the BLAST pairwise alignments and Blast Tree View tool. The Max Seq Difference is set as 0.75.
